# Pediatric Catecholaminergic Polymorphic Ventricular Tachycardia: A Translational Perspective for the Clinician-Scientist

**DOI:** 10.3390/ijms22179293

**Published:** 2021-08-27

**Authors:** Dania Kallas, Avani Lamba, Thomas M. Roston, Alia Arslanova, Sonia Franciosi, Glen F. Tibbits, Shubhayan Sanatani

**Affiliations:** 1 British Columbia Children’s Hospital Heart Center, 1F9-4480 Oak St., Vancouver, BC V6H 3V4, Canada; dania.kallas@bcchr.ca (D.K.); avani.lamba@bcchr.ca (A.L.); rostontm@alumni.ubc.ca (T.M.R.); sonia.franciosi@cw.bc.ca (S.F.); 2 Clinician-Investigator Program, University of British Columbia, 2016-1874 East Mall, Vancouver, BC V6T 1Z1, Canada; 3 Cellular and Regenerative Medicine Centre, British Columbia Children’s Hospital Research Institute, 938 W 28th Ave, Vancouver, BC V5Z 4H4, Canada; arslanov@sfu.ca (A.A.); tibbits@sfu.ca (G.F.T.); 4 Molecular Cardiac Physiology Group, Department of Biomedical Physiology and Kinesiology, Simon Fraser University, 8888 University Dr., Burnaby, BC V5A 1S6, Canada

**Keywords:** ventricular tachycardia, sudden cardiac death, pediatric electrophysiology, catecholaminergic polymorphic ventricular tachycardia, inherited arrhythmia, primary electrical disease, ryanodine receptor

## Abstract

Catecholaminergic polymorphic ventricular tachycardia (CPVT) is a rare and potentially lethal inherited arrhythmia disease characterized by exercise or emotion-induced bidirectional or polymorphic ventricular tachyarrhythmias. The median age of disease onset is reported to be approximately 10 years of age. The majority of CPVT patients have pathogenic variants in the gene encoding the cardiac ryanodine receptor, or calsequestrin 2. These lead to mishandling of calcium in cardiomyocytes resulting in after-depolarizations, and ventricular arrhythmias. Disease severity is particularly pronounced in younger individuals who usually present with cardiac arrest and arrhythmic syncope. Risk stratification is imprecise and long-term prognosis on therapy is unknown despite decades of research focused on pediatric CPVT populations. The purpose of this review is to summarize contemporary data on pediatric CPVT, highlight knowledge gaps and present future research directions for the clinician-scientist to address.

## 1. Introduction

Catecholaminergic polymorphic ventricular tachycardia (CPVT) is a rare and potentially lethal cardiac ion channelopathy associated with a normal resting electrocardiogram (ECG) and normal cardiac structure and function [1,2,3,4]. CPVT is characterized by exercise or emotion-mediated polymorphic or bidirectional ventricular tachycardia (VT), which may lead to syncope and sudden cardiac death (SCD) [5]. The prevalence of CPVT is reported to be 1:10,000 [6], however, this is an estimate lacking validation. CPVT is most associated with autosomal dominant gain-of-function variants in the *RYR2*-encoded cardiac ryanodine receptor-2 (RyR2) and recessive variants in *CASQ2*-encoded calsequestrin-2 (CASQ2), both of which affect intracellular Ca^2+^ (calcium) handling properties upon adrenergic stimulation and have a potential to trigger delayed after-depolarizations (DADs) and arrhythmias [7,8].

ß-blockers are the first line therapy, though breakthrough cardiac events often occur despite drug adherence [9,10,11]. A previous study reported that around 25% of children experienced syncope or cardiac arrest despite ß-blocker treatment [12]. In patients with recurrent arrhythmias despite ß-blockers, flecainide should be added [13,14,15,16]. Implantable cardioverter defibrillators (ICDs) are indicated for CPVT patients who have suffered cardiac arrest or have recurrent life-threatening VTs on maximal medical therapy [6]. However, ICDs have been associated with high rates of inappropriate shocks and complications, probably due to catecholamine release after an initial painful shock [17]. Left cardiac sympathetic denervation (LCSD) is another therapeutic strategy for refractory CPVT, including those with ICDs in situ [18].

Data about individual prognosis remains difficult to determine due to the broad spectrum of CPVT phenotypes described in previous reports. As many as 30% of CPVT patients have been reported to experience sudden unexpected death (SUD) by the age of 30 years [1]. Patients who are first to receive a diagnosis in their family (index case or proband) often have more severe phenotypes and are at greater risk of cardiac events [19,20]. In addition, children with CPVT typically present with more severe phenotypes compared to adults and are more vulnerable to the occurrence of sudden death [9]. This review will address pediatric CPVT specifically, by discussing genotypic advances, pathophysiology, clinical presentation, diagnoses and contemporary therapeutic options in this cohort.

## 2. History of CPVT

**Figure 1:**
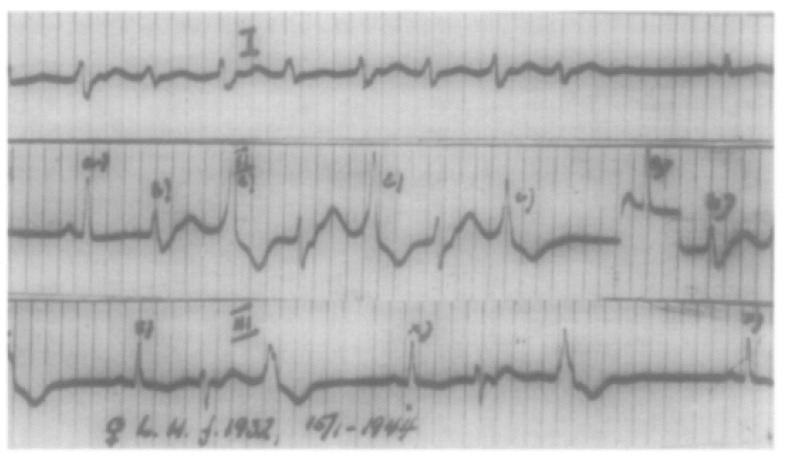
Historic electrocardiogram recording showing frequent ventricular extrasystole in 1944. Image obtained with permission from: Berg, K. J. (1960). Multifocal ventricular extrasytoles with Adams–Stokes syndrome in siblings. *American Heart Journal*, 60(6), 965–970.

CPVT was likely first described over 60 years ago by Berg [21] (Figure 1) and subsequently by Reid et al. in 1975 [22]. These reports described childhood-onset emotion and exertion induced ventricular extrasystoles in the absence of structural heart abnormalities. Reid et al. recognized that CPVT was probably familial in nature. The first longitudinal study describing CPVT as a distinct inherited cardiac disease was published in 1995 by Leenhardt et al. [1]. The genetic basis of CPVT was reported in 2001, with autosomal dominant variants in *RYR2* [7,23] and recessive variants *CASQ2* identified as causes of the disease [8].

## 3. Genetics

### 3.1. Ryanodine Receptor 2

A CPVT phenotype was first mapped to a locus on chromosome 1q42-q43 (Table 1) [2]. Subsequent studies identified missense variants in the sarcoplasmic reticulum (SR) Ca^2+^ release channel gene, *RYR2,* mapped on this disease locus [7,23]. RyR2, a large tetrameric ion channel, is involved in excitation–contraction (E-C) coupling in cardiac myocytes and plays an important role in Ca^2+^ homeostasis within the cardiac tissue [24]. Gain of function (GOF) variants in *RYR2* are associated with an autosomal dominant form of CPVT [7], and are attributed to 60% of cases [7].

### 3.2. Calsequestrin 2

In 2001, Lahat et al. found missense variants in highly conserved regions of another gene involved in the regulation of Ca^2+^ homeostasis of the heart, *CASQ2*, causing an autosomal recessive form of CPVT in seven Bedouin families [8]. *CASQ2* has historically been associated with an autosomal recessive form of CPVT, however autosomal dominant inheritance has also been described in isolated studies and further confirmed in a recent international multi-centre study on *CASQ2* inheritance patterns [25,29,30]. Approximately 2–5% of CPVT cases are attributed to *CASQ2* variants. However, given that heterozygote *CASQ2* patients may also have a CPVT phenotype, the true prevalence of *CASQ2* associated CPVT is debatable [25]. Pathogenic variants in the *RYR2* and *CASQ2* genes underlie CPVT1 and CPVT2, respectively, with CPVT2 patients typically presenting at a younger age [8,25,31].

### 3.3. Trans-2,3-enoyl-CoA Reductase-like

Several additional genes have been linked to CPVT. A CPVT phenotype associated with an autosomal recessive mode of inheritance, mapped to chromosome locus 7p22–p14, was first described in a consanguineous Arabic family by Bhuiyan et al. in 2007 [32]. This disease form was recently attributed to gene variants in trans-2,3-enoyl-CoA reductase-like (*TECRL*) encoding a protein expressed in the endoplasmic reticulum of myocardial cells, belonging to the steroid 5-alpha reductase family. Patients with variants in *TECRL* comprise <1% of CPVT cases and the molecular mechanism of disease in these patients is unestablished. This is a highly lethal form of CPVT, with patients first presenting symptoms during childhood, with a recent multicentre study reporting a median age of symptom onset at 8 years of age [33].

### 3.4. Calmodulin and Triadin

Variants in *CALM1*-encoded calmodulin, a Ca^2+^ binding protein, and *TRDN*-encoded Triadin, a cardiac Ca^2+^ release complex protein, have also been implicated in atypical CPVT and are associated with 1–2% of CPVT cases [27,28]. Phenotypes associated with these genes are more complex and variable than classic CPVT. Other mimickers of CPVT also exist. A minority of patients (<1%) with phenotypic features of CPVT have also been found to be heterozygous for *ANK2, SCN5A,* or *KCNJ2* variants [34,35,36,37]. None of these genes cause CPVT in isolation, and instead lead to complex overlapping forms of long QT syndrome (LQTS) and ventricular ectopy [38,39].

### 3.5. Expressivity and Penetrance

It is increasingly recognized that CPVT associated genetic variants can result in incomplete penetrance and variable expressivity. Various studies have described marked phenotypic diversity amongst young relatives carrying *RYR2* variants diagnosed through cascade screening. [5] [19,40]. Variable expressivity of the CPVT phenotype could possibly be explained by polygenic influences that cause certain CPVT patients to be more susceptible to life-threatening arrhythmic events. In other autosomal dominant inherited cardiac diseases, like hypertrophic cardiomyopathy, Brugada syndrome and LQTS, genetic modifier research is more mature [41,42,43,44]. Conversely, no modifiers have been identified in CPVT to date [19,45]. A minority of pediatric CPVT patients remain genetically elusive [31] making gene discovery in this subset of patients necessary. Genome wide association studies (GWAS) may be a beneficial next step in identifying disease susceptibility variants. A recent transethnic GWAS study in LQTS utilized polygenic score analyses and demonstrated polygenic influence in expression of phenotype in genotype negative patients [46].

## 4. Pathophysiology

### 4.1. Mechanisms of Intracellular Ca^2+^ Regulation

**Figure 2:**
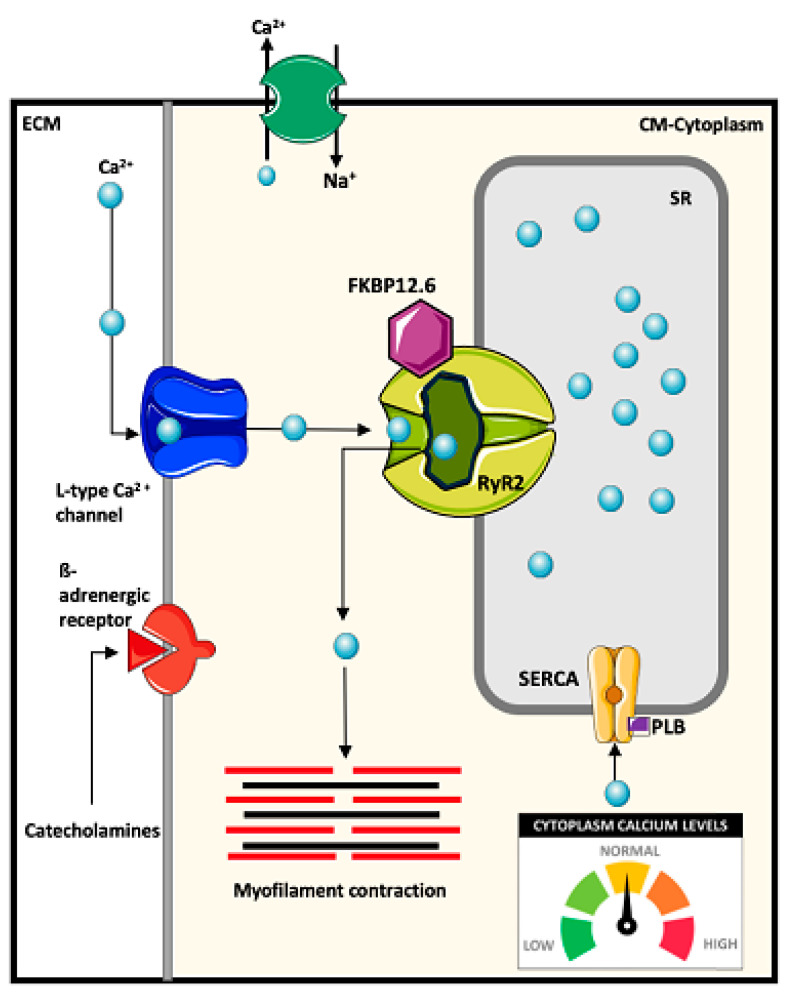
Ca^2+^ induced Ca^2+^ release mechanism: The cardiac action potential leads to membrane depolarization, thereby opening L-type Ca^2+^ channels (Ca_V1.2_). Ca^2+^ in the cytosol then binds to the ryanodine receptor 2 (RyR2) leading to CICR from the sarcoplasmic reticulum (SR) lumen into the cytosol. During systole, the cytosolic Ca^2+^ then binds to troponin C which leads to myocardial contraction. Ca^2+^ in the cytoplasm is returned to the SR via the sarco/endoplasmic reticulum Ca^2+^-ATPase (SERCA2a) and its regulatory protein phospholamban (PLB). CM = cardiomyocyte; ECM = extracellular matrix. Image adapted from Priori, S. G., and Chen, S. W. (2011), *Circulation Research,* 108(7), 871–883 [49].

CPVT-associated genes encode proteins vital to the regulation of Ca^2+^ in cardiomyocytes. During the cardiac E-C coupling, a wave of depolarization originating from the influx of Na^+^ (sodium) through the Na^+^ channels (Na_V1.5_), traverses through the sarcolemma and reaches the t-tubules, which causes opening of the L-type Ca^2+^ channels (Ca_V1.2_ and also known as dihydropyridine receptors). This facilitates subsequent influx of Ca^2+^ into the cytosol. An increase in cytosolic Ca^2+^ triggers Ca^2+^ release from the SR (the Ca^2+^ storage unit of the cardiac myocyte) through a process known as Ca^2+^-induced Ca^2+^ release (CICR) as a result of RyR2 activation by cytosolic Ca^2+^ (Figure 2) [47]. RyR2 Ca^2+^ release is facilitated by CASQ2 (a Ca^2+^ buffering protein within the SR regulating the levels of free Ca^2+^ in the SR), triadin and junctin (both mediate the interaction between Ca^2+^ handling proteins, CASQ2 and RyR2) forming the SR Ca^2+^ release complex. Together, they also confer RyR2 responsiveness to luminal Ca^2+^ [48].

During systole, the cytosolic Ca^2+^ then binds to troponin C of the troponin complex facilitating myocardial contraction [50,51]. E-C coupling can also be influenced by sympathetic nervous system (i.e., exercise or stress) which promotes catecholamine release and subsequent activation of protein kinase A (PKA)-mediated pathway as a result of ß_1_-adrenergic stimulation. PKA is capable of phosphorylating several key regulatory and accessory proteins modulating cardiac contractility through regulation of intracellular Ca^2+^ [52]. These target proteins include: (1) L-type Ca^2+^ channels, increasing Ca^2+^ influx and eventually CICR from the SR [53]; (2) RyR2, enhancing SR Ca^2+^ release; (3) phospholamban (PLB), promoting Ca^2+^ reuptake back into the SR through disinhibition of sarco/endoplasmic reticulum Ca^2+^-ATPase (SERCA2a) [53,54]; or (4) troponin I and myosin-binding protein C, improving myofilament Ca^2+^ sensitivity, myocardial relaxation rate and force generation [53,55,56]. During diastole, myocardial relaxation is induced by cycling the cytosolic Ca^2+^ through two primary mechanisms involving SERCA2a which pumps Ca^2+^ back into SR and the Na^+^/Ca^2+^ exchanger (NCX) which extrudes Ca^2+^ out of the cell [57,58].

In addition to CICR-mediated SR Ca^2+^ release, the spontaneous release of Ca^2+^ from the SR can also be caused by store overload-induced Ca^2+^ release (SOICR) which can trigger Ca^2+^ oscillations [49]. Disruption in intracellular Ca^2+^ handling affecting these Ca^2+^ release pathways can facilitate the onset of DADs. If the membrane depolarization produced by DADs is large enough, it can lead to repeated premature and uncoordinated activation of cardiac tissue [50,51].

### 4.2. Ryanodine Receptor-2 Associated Arrhythmogenesis

Gain of function variants in *RYR2* lead to altered functional properties making the channel prone to spontaneous diastolic SR Ca^2+^ leak. This effect is further exacerbated during the episodes of sympathetic activation (i.e., exercise, stress) due to phosphorylation of several key Ca^2+^ handling proteins mediated by the ß_1_-adrenergic pathway. The resulting aberrant SR Ca^2+^ release causes a local rise in Ca^2+^ which activates NCX to drive Ca^2+^ extrusion in exchange for Na^+^ influx. This generates a transient inward current that promotes the onset of DADs, which if large enough to reach a threshold for Na^+^ channel activation, can subsequently initiate action potentials and the onset of VT [49,59].

**Figure 3:**
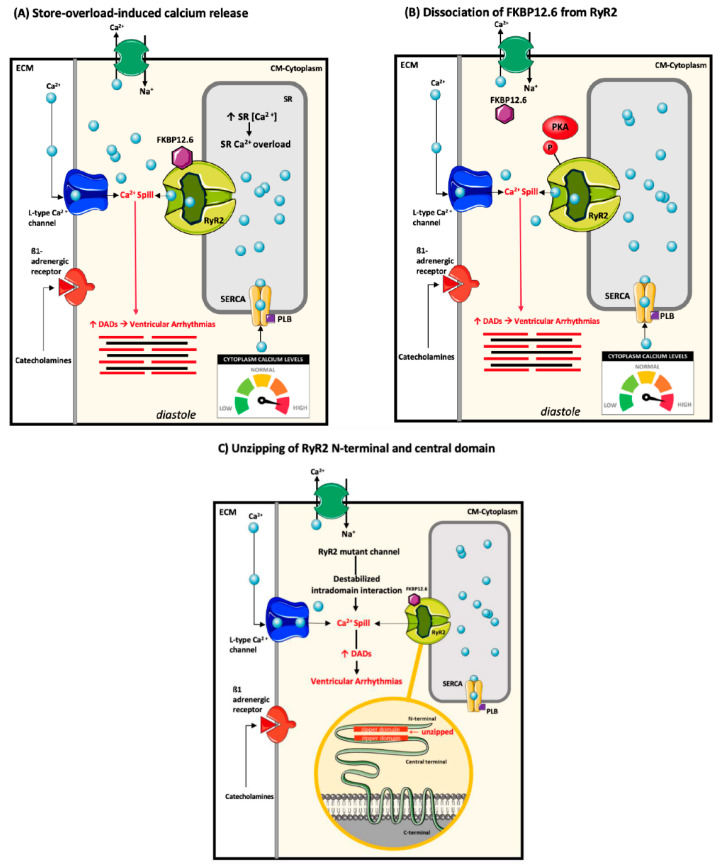
Proposed mechanisms of *RYR2*-associated CPVT arrhythmogenesis: (**A**) Store overload-induced Ca^2+^ release: RyR2 mutant channels may exhibit an increased sensitivity to the luminal Ca^2+^ in the SR by decreasing the threshold needed to activate it, leading to increased Ca^2+^ release from the SR during diastole. This leads to delayed afterdepolarizations (DADs) and thus, uncoordinated activation of cardiac tissue and triggered ventricular arrhythmias. (**B**) FKBP12.6 dissociation from the RyR2-complex: FKBP12.6 is phosphorylated by protein kinase A, leading to its dissociation from mutant RyR2, increasing diastolic Ca^2+^ release and consequent contractility. (**C**) Unzipping of the N-terminal and central terminal of the RyR2 protein: N-terminal and the central domain of wildtype RyR2 interact to form a tight intramolecular structure or a “zip” to stabilize the channel. However, RyR2 mutant channels cause weak intramolecular domain interaction resulting in Ca^2+^ spill. P= phosphate group; Other abbreviations as indicated in Figure 2. Image adapted from Wleklinski, M. J., Kannankeril, P. J., and Knollmann, B. C. (2020). *The Journal of Physiology*, *598*(14), 2817–2834 [60].

Despite the commonality in the mechanism that leads to DADs underlying CPVT arrhythmogenesis, several hypotheses, likely influenced by the location of the *RYR2* variants and their effect on structure, gating, and regulation of the channel, have been outlined to converge to this conclusion (Figure 3) [60,61]. A hypothesis that has been well documented by Chen and colleagues suggests that *RYR2* variants enhance channel’s sensitivity to the luminal Ca^2+^ concentration reducing the threshold for spontaneous SR Ca^2+^ through a SOICR-mediated mechanism (Figure 3A) [49,62,63,64]. Reduced SOICR threshold promotes activation of RyR2 at lower SR Ca^2+^ amounts increasing susceptibility to triggered DADs, and these effects are even further exacerbated during β-adrenergic stimulation [49]. A second hypothesis underlying *RYR2*-dependent CPVT proposes that *RYR2* variants cause an impaired binding affinity of the FKBP12.6, a protein involved in stabilizing a closed state of the RyR2 channel during diastole, to the RyR2 monomers (Figure 3B) [65]. This particularly impacts RyR2 function during PKA-mediated phosphorylation which causes further dissociation of FKBP12.6 from RyR2 increasing its open probability state which, thereby, promotes diastolic SR Ca^2+^ leak [65]. This mechanism has been challenged by Xiao et al., who found that loss of FKBP12.6 has no effect on conduction or activation of RyR2, nor does it affect the susceptibility to stress-induced ventricular arrythmias [66]. A third hypothesis proposes that impaired RyR2 interdomain interaction between the N-terminal and central domains, as a result of *RYR2* variants, result in folding abnormalities that destabilize the functional behavior of the channel (Figure 3C) [67]. Normally, the domains interact to form a tight intramolecular structure, “zipping” the channel in a closed conformation and stabilizing it [68]. *RYR2* variants affecting these domains have the ability to weaken intramolecular interactions of the N-terminal/central domain pair which contributes to Ca^2+^ leak through RyR2 and initiation of DADs [63,68,69,70,71].

### 4.3. Calsequestrin-2 Associated Arrhythmogenesis

CASQ2 is a high capacity and low affinity Ca^2+^ binding protein. It forms dynamic Ca^2+^-dependent linear polymers through the binding of Ca^2+^ in electronegative pockets within CASQ2 dimers, leading to dimer cross bridging [72,73]. Variants in *CASQ2* leads to a reduction in this protein’s Ca^2+^ binding capacity [72]. *CASQ2* variants can lead to various protein structural abnormalities, ranging from CASQ2 monomers that are unable to dimerize to disrupted hydrophobic cores in protein domains resulting in formation of Ca^2+^ desensitized high molecular aggregates [72]. A recent study on pathogenic heterozygous *CASQ2* variants showed that mutant proteins displayed filamentation defects, with one variant failing to dimerize.

### 4.4. TRDN, CALM1, and TECRL Associated Arrhythmogenesis

Pathogenic variants in *TRDN*, *CALM1,* and *TECRL* are rare causes of CPVT3, CPVT4, and CPVT5, respectively. Autosomal recessive *TRDN* variants were found to be associated with intracellular retention and protein degradation of mutant Triadin both in cell culture and mouse models [28]. Dominantly inherited *CALM* variants have been shown to cause Ca^2+^ binding defects as well as disrupted RyR2-Calmodulin protein interactions [27]. *TECRL* has been shown to play a role in intracellular Ca^2+^ homeostasis, with mutant human induced pluripotent stem cell-derived cardiomyocytes (hiPSC-CMs) showing elevated diastolic Ca^2+^ levels, decreased SERCA and NCX activities, and prolonged action potentials compared to control hiPSC-CMs [74].

## 5. Clinical Presentation

Most CPVT patients experience cardiac events during exercise or emotional stress. Cohort studies of pediatric CPVT patients are presented in Table 2. Life-threatening cardiac events during wakeful and normal daily activities have also been reported, possibly due to anxiety, stress, or other non-exertional psychological stimuli [31]. Patients typically present with palpitations, syncope, arrhythmia-induced seizures, resuscitated cardiac arrest, or SCD. Symptoms in CPVT are usually due to polymorphic VT, bidirectional VT, or ventricular fibrillation (VF), but may also be caused by atrial arrhythmias [75]. Sy et al. reported a bimodal age distribution in CPVT onset, with a third of patients presenting before the age of 21 [11]. They also found that patients presenting at an older age were less likely to have an *RYR2* variant [11]. By age 10, about 35% of patients are symptomatic, and this rises to 72% by 21 years of age, indicating that CPVT is a predominantly pediatric-onset condition [9]. SCD in a relative <40 years of age has been reported in 74% of asymptomatic children evaluated as part of family screening [12].

CPVT is relatively uncommon in infants, with the youngest age of symptom onset reported as 2 years of age [10], however sudden infant death syndrome potentially caused by CPVT has previously been reported in two infants harbouring *RYR2* gain of function missense variants [80]. Children can be misdiagnosed as epileptic prior to a CPVT diagnosis, due to a clinical presentation of seizures and exertion or emotion induced syncope [1,9,81]. Interestingly, *RYR2* variants have also been linked to epilepsy [82,83]. Patients can experience a delay in diagnosis due to arrhythmogenic syncope being misdiagnosed as benign vasovagal events. CPVT may also be misdiagnosed as LQTS since patients may present with similar symptoms, inclusive of exercise-induced syncope, and SUD at similar ages [84,85]. The failure to perform an EST for the evaluation of patients with exertion or emotional induced symptoms or the misinterpretation of an EST can lead to missed or delayed diagnoses [86]. Although, polymorphic VT (occurring in the setting of prolonged QT in LQTS) are features of both diseases, LQTS does not induce progressive ventricular ectopy (monomorphic premature ventricular complexes (PVCs) → PVCs in bigeminy or couplets→ bidirectional or polymorphic VT) on a treadmill test. Further clinical investigation including resting ECG, inclusion of EST in immediate post sudden cardiac arrest evaluation, and genetic testing can help with the differentiation of both diseases [84]. Andersen–Tawil Syndrome (ATS), a *KCNJ2*-associated disease, may be mistaken for CPVT due to the presence of ventricular ectopy and bidirectional VT during exercise stress testing in both conditions. However, the ATS phenotype is also accompanied by characteristic orthopedic and facial deformities which is a clear differentiating factor from CPVT [39], especially in the absence of a prolonged QT interval and U-waves on a resting ECG [87].

In addition to ventricular arrhythmias, a proportion of CPVT cases have supraventricular arrhythmias [19]. Based on one report of CPVT patients, 38% of CPVT cases were associated with atrial fibrillation or atrial flutter [75]. In another report, 19% of patients were described to have atrial fibrillation and junctional tachycardia [1]. CPVT patients may also have sinus node dysfunction and inducible atrial arrhythmias, suggesting that the disease phenotype is not limited to ventricular arrhythmias but might also involve the sinus node and atrium [75].

## 6. Diagnosis

In CPVT, the resting ECG often shows normal sinus rhythm or sinus bradycardia without conduction abnormalities [19]. Cardiac imaging tests such as echocardiograms and cardiac magnetic resonance imaging are also normal [6]. Therefore, CPVT should be considered in the differential diagnosis in patients with unexplained life-threatening arrhythmias with a normal resting ECG and imaging [88]. In addition, the expert consensus recommendations state that one of the following should be present to make a CPVT diagnosis [6]:

- An unexplained catecholamine-induced bidirectional VT or polymorphic ventricular premature beats (VPBs), or VT in an individual younger than 40 years;
- A patient (index case or family member) with a pathogenic variant in a CPVT-related gene (see Genetic Etiology for details); and
- Family members of a CPVT index case with a normal heart who manifest exercise-induced PVCs or bidirectional or polymorphic VT.

An exercise stress test (EST) is the current standard to diagnose CPVT. Ventricular arrhythmias are provoked in 80% of symptomatic probands, although mild forms of ventricular ectopy may be overlooked [1,7,10]. Typically, PVCs occur around a heart rate of 100–120 beats per minute, and progress to bigeminy, couplets, and non-sustained polymorphic or bidirectional VT as heart rate increases [11]. Ventricular arrhythmias in CPVT are often initiated in the right ventricular outflow tract in pediatric patients [10]. A normal or near normal EST does not reliably refute the diagnosis of CPVT, especially when the pre-test probability of the condition is moderate to high [89]. Van der Werf et al. reported that 50% of patients who were unaffected at their first cardiac examination developed a phenotype during re-evaluation at a median follow-up of 1.6 years [19]. We recently reported a new EST “Burst” protocol that unmasked new or more complex arrhythmias in children not identified by standard EST alone [90]. This is based on the concept that a sudden sprint is more likely to induce CPVT than a gradual graded protocol, like the Bruce protocol. In patients who are too young to run on a treadmill or not able to reach sufficient heart rates during exercise, an ambulatory ECG monitor or a Holter monitor can be diagnostic [91]. Intravenous epinephrine infusion test, which mimics endogenous catecholamine release, has also been proposed as an alternative to EST for diagnosing CPVT. However, a study of 81 CPVT patients showed that in comparison to EST, pharmacological provocation with epinephrine only had a 28% sensitivity and therefore should not be part of routine practice [92].

### Genetic Testing Guidelines in CPVT

Genetic testing has two main utilities in CPVT: (1) to confirm a clinical diagnosis; and (2) to inform cascade family screening. Importantly, since *RYR2* and *CASQ2* variants are identified in around 60% of CPVT cases [12,93], and the background rate of rare benign variation in *RYR2* approaches 3% [94,95], genetic testing should not be used to rule out the diagnosis when clinical suspicion exists or rule it in when the variant is not definitively pathogenic. The most recent expert consensus statement on genetic testing guidelines in channelopathies recommend comprehensive *RYR2* and *CASQ2* targeted CPVT genetic testing for patients with a clinical index of suspicion for CPVT based on clinical history, family history and development of electrocardiographic phenotype with provocative testing. Additionally, variant-specific testing in relatives of index cases with an identified CPVT-causative variant, is also recommended [96]. Approximately 8% of children with CPVT will have ≥2 variants, which poses challenges with risk stratification, highlighting the importance of family based genetic counselling [45]. Therefore, ordering broader genetic panels is unlikely to be helpful unless the phenotype is overlapping or syndromic features are present since variant interpretation becomes challenging.

*RYR2*-associated CPVT variants often cluster in four mutational hotspots reported by Priori and Chen based on aggregate data from the literature: Hotspot I (amino acids 44-466; ~18% of mutations) located in the N-terminus domain (amino acid 77-446); Hotspot II (2246-2534; ~19% of mutations) located in the central domain (2246-2534); Hotspot III (3778-4201; ~22% of mutations) and Hotspot IV (4497-4959; ~22% of mutations) located in the C-terminus domain (3778-4959) [49,97]. Guidelines on the utility of genetic testing in CPVT do not fully address all the important nuances of sequencing nor the interpretation of a variant. A newly devised “phenotype-enhanced” scoring system for *RYR2* variants has recently been described, which factors the pre-test probability of CPVT into the assignment of pathogenicity [95]. In addition, more than 24 pathogenic *CASQ2* variants associated with CPVT have been identified to date [79]. Consanguineous family members are typically tested for *CASQ2* variants. Pathogenic *CASQ2* variants have not only been reported in an autosomal recessive pattern, but also in heterozygotes with an autosomal dominant inheritance pattern [25]. The putative mechanism of *CASQ2* heterozygous CPVT is a failure of back-to-back dimerization of mutant CASQ2, an effect that may be variant specific, and result in generally mild CPVT [25].

## 7. Therapy and Management

### 7.1. β-Blockers

β-blockers are the mainstay of treatment in CPVT patients [6]. β-blockers act by inhibiting β-adrenergic mediated-activation of RyR2 channels. β-blockers alone may offer incomplete protection. A meta-analysis of 11 studies comprising 403 mostly young CPVT patients, reported β-blocker use in 88% of symptomatic patients with an eight-year arrhythmic (syncope, ACA, SCD), near-fatal (ACA, SCD), and fatal event rates of 37.2%, 15.3%, and 6.4%, respectively [97]. Although β-blockers have been effective for the majority of patients, the arrhythmic event rate on β-blocker remains significant. Therefore, a stepwise addition of other therapeutic options is recommended when β-blockers fail [97]. Another pediatric cohort study found that arrhythmic syncope or cardiac arrest while on β-blockers occurred in 13% of CPVT patients [31]. Non-adherence is a major problem, especially in children [12]. Nadolol, a non-selective β-blocker, is preferred and should be prescribed at a dosage of at least 1–2 mg/kg (divided into two doses/day). Hayashi et al. were the first to describe lower cardiac event rates in patients treated with nadolol versus other β-blockers among patients diagnosed at a median age of 15±10 years [9]. Another study reported decreased incidence and severity of ventricular arrhythmias in patients during treatment with nadolol relative to β_1_-selective β-blockers [98]. Non-selective β-blockers should be prescribed to all CPVT patients except for those who have contraindications or intolerance, irrespective of sinus node dysfunction.

### 7.2. Flecainide

When β-blockers are ineffective at suppressing ventricular ectopy, flecainide is usually prescribed as combination therapy. Flecainide is a class 1c antiarrhythmic and potent Na^+^ channel blocker and is prescribed in divided doses of 100–300 mg/day. Flecainide in combination with β-blockers has been shown to reduce exercise-induced ventricular arrhythmias in children and adults with breakthrough events on β-blockers alone [13,14]. Another study showed that flecainide prevented recurring ICD shocks in addition to exercise-induced ventricular arrhythmias in patients with *CASQ2* associated CPVT [16]. Padfield et al. described a small series suggesting that flecainide monotherapy can be considered in low-risk CPVT patients intolerant of β-blockade, however further data are needed to confirm the protectiveness of this approach [99]. The mechanism of action of flecainide in CPVT remains debated in the literature. An early mechanistic study found that flecainide directly interacts with RyR2 and inhibits its activity [15]. This theory is supported by evidence presented in a recent study by Kryshtal and colleagues [59]. However, Bannister et al. suggested that flecainide does not have a direct interaction with RyR2, rather it affects the cytoplasmic proteins associated with RyR2 [100].

### 7.3. Left Cardiac Sympathetic Denervation

LCSD is an alternative therapy in patients when β-blockers are poorly tolerated or fail to prevent recurrent syncope, cardiac arrest or ICD shocks [101]. LCSD involves removal of the lower half of the left stellate ganglion (T1), along with the thoracic ganglia T2 through T4, in order to reduce the sensitivity of the heart to catecholamines [102]. The reduction in the release of the catecholamine norepinephrine by sympathetic neurons in ventricular myocytes contributes to LCSD’s antiarrhythmic and antifibrillatory effects [102]. In a large cohort comprising 63 patients, LCSD was associated with a decrease in cardiac events and a decrease in the rate of ICD shocks [18]. LCSD is a promising therapy in CPVT, although its lack of accessibility worldwide will remain a limitation to its widespread implementation. Further data on indication and long-term outcomes would be helpful.

### 7.4. Implantable Cardioverter Defibrillators

Current North American and European guidelines recommend that an ICD be reserved for CPVT patients with prior SCA, or those with refractory ventricular arrhythmias on combination pharmacological therapy [103,104]. ICDs may be considered in children who experience breakthrough arrhythmic events or sustained VT or VF despite antiarrhythmic treatment [6]. However, ICDs can be ineffective and pro-arrhythmic in young CPVT patients, with deaths being reported despite appropriate ICD therapy [105,106,107]. Appropriate shocks have been reported to be ineffective at terminating sustained VT [108]. Implantation of an ICD poses a technical challenge in pediatric patients given the high rate of device related complications and inappropriate shocks [108,109], which may be a consequence of supraventricular tachycardia such as atrial arrhythmias [9,11]. Both appropriate and inappropriate discharges can cause catecholamine surges which can lead to electrical storm and potentially life-threatening arrhythmias [110]. Other challenges with implanting ICDs in children include optimal programming, the psychological trauma of repeated shocks, high risk of inappropriate therapies due to atrial arrhythmias, and life-long burden of mechanical and infectious complication risk. Therefore, determining which patients benefit from ICDs in CPVT is an urgent area of need.

### 7.5. Lifestyle Management: Sport Participation

Given that adverse cardiac events often manifest during adrenergic stress, patients and families must be advised on sports participation. The European Society of Cardiology guidelines for management of patients with SCD recommend that CPVT patients avoid competitive sports, strenuous exercise, and exposure to stressful environments to avoid the consequences of adrenergic stimulation [103]. These restrictions can have a psychological impact on children. In fact, a study on competitive sports participation in CPVT patients found no significant difference in incidence of cardiac events between athletes and non-athletes, indicating that the risk of sports participation may be acceptable among informed athletes adhering to guideline recommended therapy [111].

### 7.6. Other Therapeutic Interventions

Another intervention which has been studied in CPVT but is not part of any routine therapeutic regiment is dantrolene. Dantrolene, commonly used for malignant hyperthermia, affects Ca^2+^ flux across the SR and cardiomyocyte function in failing hearts. In CPVT mouse models, dantrolene was effective in inhibiting inducible VT, and reduced diastolic Ca^2+^ sparks and DADs [112] by correcting defective inter-domain interactions between the N-terminal and central domain of RyR2 [112,113,114]. A study by Penttinen et al. showed that dantrolene reduced the number of PVCs by 75% in four of six patients with variants in the N-terminal/central terminal but had no effect in patients with variants in the transmembrane domain [115]. Although dantrolene has shown to be effective in some patients, data is limited and further investigation is warranted before this therapy can be recommended in CPVT.

There are few acute treatment options which may be considered in CPVT, however these typically vary from patient to patient. Standard defibrillation therapy (either an automated external defibrillator or ICD) can be fatal due to a potential surge in catecholamines which can lead to generation of DADs and consequent arrhythmia [105,108]. Therefore, sedation, intubation, and stabilization of the patient’s rhythm is of paramount importance. In some cases, a magnet can be used to reduce the risk of ICD storms [116]. Intravenous β-blockers are another therapeutic option, which can effectively resolve arrhythmias in most patients. IV-esmolol is a common therapeutic option due to its intravenous formulation and short half-life [116]. Flecainide may also be used intravenously, however its efficacy in the acute setting is largely unknown. Finally, propranolol has also been shown to be efficacious in the management of acute VT [117]. Overall, these treatment options stem from CPVT’s disease mechanism and extrapolation from chronic therapies, yet no data is available to support their use in the acute setting.

Gene therapy is an exciting novel area of research on CPVT therapeutics, albeit in its infancy. A recent study by Bezzerides et al. showed that adeno-associated virus mediated CaMKII peptide inhibitor delivery to the heart effectively suppressed CPVT-associated arrhythmias in a murine CPVT model [118]. These inhibitory peptides also successfully reversed arrhythmias in hiPSC-CM models of CPVT [118]. Additionally, CRISPR/Cas9 gene editing has also been used in hiPSC CPVT disease models to evaluate drug sensitivities of pathogenic variants [119,120].

## 8. Conclusions

CPVT is a rare and lethal inherited arrhythmia disease characterized by VT in the absence of structural heart disease often presenting in childhood. The knowledge surrounding disease mechanism, diagnosis, treatment, and prognosis has improved over the last two decades, but several gaps remain. These include ongoing questions surrounding the primary mechanism of arrhythmia and target of flecainide, safety of physical activity while on therapy, risk stratification to inform ICD and ancillary therapy decision-making, and the potential role of genetic modifiers in explaining variability in disease penetrance and expressivity, especially in pediatric CPVT. Centers leading large registries need to develop translational science opportunities for the clinician-scientist aimed at addressing the interplay between genotype and these gaps.

## Figures and Tables

**Table 1:** Genetic subtypes of CPVT.

CPVT Subtype	Gene	Protein Name	Chromosome Locus	Inheritance Pattern	Proportion of Cases Associated with This Gene	Year Discovered
CPVT1	*RYR2*	Ryanodine Receptor 2	1q43	Autosomal Dominant	55–60% [7]	2001
CPVT2	*CASQ2*	Calsequestrin 2	1p13.1	Autosomal Recessive/ Autosomal Dominant [25]	2–5% [8]	2001
CPVT3 *	*TECRL*	Trans-2,3-enoyl-CoA reductase-like	7p22–p14	Autosomal Recessive	<1% [26]	2016
CPVT4 *	*CALM1*	Calmodulin	14q32.11	Autosomal Dominant	<1% [27]	2012
CPVT5 *	*TRDN*	Triadin	6q22.31	Autosomal Recessive	1–2% [28]	2012

* Usually with other distinctive evidence of a competing inherited arrhythmia syndrome phenotype.

**Table 2:** Cohort studies of pediatric CPVT patients.

Author	Year Published	CPVT Cases (n)		Mean/Median Age of Symptom-Onset (Years)	Cardiac Events at Follow-Up ^†^	Conclusions/Significance
		Proband	Family Member			
Leenhardt et al. [1]	1995	20	1	7.8 ± 4.0	2 SCDs over a mean of 7 years	First large description of CPVT as adrenergic-induced lethal tachyarrhythmias in the absence of structural heart disease
Swan et al. [2]	1999	14 (in 2 families)		21 ± 10	1 patient with syncope and cardiac arrest over 8 ± 6 years	Arrhythmia disease mapped to chromosome 1q42-q43.
Lahat et al. [8]	2001	13 (in 7 families)		6 ± 3	1 SCD over 40 months	First *CASQ2-*associated autosomal recessive CPVT case reported.
Bauce et al. [4]	2002	43 (in 8 families)		Not reported	No events during follow-up	Genetic screening is important for early diagnosis of asymptomatic carriers.
Priori et al. [3]	2002	30	9	8 ± 2, *RYR2*-associated cases; 20 ± 12, Non-genotyped cases	Not reported	Non-genotyped CPVT cases are often women with late-onset of symptoms. *RYR2-*associated CPVT often have an early-onset of symptoms. Men are at higher risk of cardiac events.
Sumitomo et al. [10]	2003	25	4	10.3 ± 6.1	7 (25%) SCD over mean 6.8 ± 4.9 years	Prognosis is poor.
Postma et al. [5]	2005	12	42	12, *RYR2-*associated cases	1 (8%) SCD over median of 6 years	Patients with *RYR2* variants have a significant resting sinus bradycardia.
Hayashi et al. [9]	2009	50	51	12 ± 8	27 patients over 7.9 ± 4.9 years	Risk factors for arrhythmic events include younger age at diagnosis and absence of β-blocker therapy
van der Werf et al. [19]	2012	24	116 (61 from 1 family)	Not reported	4 (22%) probands over a median of 7.8 years; 2 relatives over a period of 6.7 years	Relatives have marked phenotypic diversity and less severe phenotypes compared with probands.
Kawamura et al. [76]	2013	50	0	10.2 ± 7.3	5 (19%) experienced syncope over mean follow-up of 48 months	Penetrance of CPVT phenotype was significantly higher in patients with *RYR2* variants among Japanese CPVT patients.
Ohno et al. [77]	2015	36	0	9.7 ± 4.6 *	Not reported	Almost half of *RYR2* variants are de novo, and others are more often inherited from mothers than fathers.
Roston et al. [12]	2015	170	56	10.8 (6.8–13.2)	Not reported	CPVT can have a malignant phenotype and lengthy delay to diagnosis, probands are typically severely affected.
Jiang et al. [78]	2018	12		8.4 ± 3.2	1 SCD over 0.92 ± 0.8 years	Severe delay to diagnosis and misdiagnosis of CPVT are not uncommon in China.
Li et al. [79]	2019	5	2	6.2 ± 1.3	No events over mean follow-up of 16.5 months *	First systematic study to examine CASQ2-CPVT in Chinese children identifying three novel variants.
Ng et al. [25]	2020	36	76	7.9±3.3, probands only	Not reported	Patients with pathogenic CASQ2 heterozygous variants may manifest CPVT phenotype.
Kallas et al. [20]	2021	106	27	11 (7–13.5)	44 (33%) inclusive of a 3% mortality rate over 6-years (3–11) after time of symptom-onset	Proband status, but not age of symptom-onset or male sex, independently predicted an earlier-onset of cardiac events.

Continuous variables were presented as mean with standard deviation (± SD), median with interquartile range (IQR) where applicable; SCD = sudden cardiac death; ^†^ Cardiac events are inclusive of cardiac arrest, arrhythmia-induced syncope and SCD during follow-up; * Calculated from data set.

## Data Availability

Not applicable.
